# AaeAP1 and AaeAP2: Novel Antimicrobial Peptides from the Venom of the Scorpion, *Androctonus aeneas*: Structural Characterisation, Molecular Cloning of Biosynthetic Precursor-Encoding cDNAs and Engineering of Analogues with Enhanced Antimicrobial and Anticancer Activities

**DOI:** 10.3390/toxins7020219

**Published:** 2015-01-23

**Authors:** Qiang Du, Xiaojuan Hou, Lei Wang, Yingqi Zhang, Xinping Xi, Hui Wang, Mei Zhou, Jinao Duan, Minjie Wei, Tianbao Chen, Chris Shaw

**Affiliations:** 1 School of Pharmaceutical Sciences, China Medical University, Shenyang 110001, China; E-Mails: qdu01@qub.ac.uk (Q.D.); xhou01@qub.ac.uk (X.H.); mjwei@hotmail.com (M.W.); 2 Natural Drug Discovery Group, School of Pharmacy, Queen’s University, Belfast BT9 7BL, Northern Ireland, UK; E-Mails: l.wang@qub.ac.uk (L.W.); xxi01@qub.ac.uk (X.X.); dja@njutcm.edu.cn (J.D.); t.chen@qub.ac.uk (T.C.); chris.shaw@qub.ac.uk (C.S.); 3 Department of Emergency Medicine, the First Hospital of Hebei Medical University, Shijiazhuang 050031, China; 4 Jiangsu Key Laboratory for Traditional Chinese Medicine (TCM) Formulae Research, Nanjing University of Chinese Medicine, Nanjing 210023, China

**Keywords:** scorpion, venom, antimicrobial peptide, molecular cloning

## Abstract

The main functions of the abundant polypeptide toxins present in scorpion venoms are the debilitation of arthropod prey or defence against predators. These effects are achieved mainly through the blocking of an array of ion channel types within the membranes of excitable cells. However, while these ion channel-blocking toxins are tightly-folded by multiple disulphide bridges between cysteine residues, there are additional groups of peptides in the venoms that are devoid of cysteine residues. These non-disulphide bridged peptides are the subject of much research interest, and among these are peptides that exhibit antimicrobial activity. Here, we describe two novel non-disulphide-bridged antimicrobial peptides that are present in the venom of the North African scorpion, *Androctonus aeneas*. The cDNAs encoding the biosynthetic precursors of both peptides were cloned from a venom-derived cDNA library using 3'- and 5'-RACE strategies. Both translated precursors contained open-reading frames of 74 amino acid residues, each encoding one copy of a putative novel nonadecapeptide, whose primary structures were FLFSLIPSVIAGLVSAIRN and FLFSLIPSAIAGLVSAIRN, respectively. Both peptides were *C*-terminally amidated. Synthetic versions of each natural peptide displayed broad-spectrum antimicrobial activities, but were devoid of antiproliferative activity against human cancer cell lines. However, synthetic analogues of each peptide, engineered for enhanced cationicity and amphipathicity, exhibited increases in antimicrobial potency and acquired antiproliferative activity against a range of human cancer cell lines. These data clearly illustrate the potential that natural peptide templates provide towards the design of synthetic analogues for therapeutic exploitation.

## 1. Introduction

The vast majority of scorpion venom toxins studied thus far function through altering the functions of cell membrane ion channels [1,2,3,4]. Ion channels play fundamental roles in the regulation of many physiological aspects of all cells and, additionally, have been implicated in the pathogenesis of many diseases [5,6]. Thus, the acquisition of the ability to disturb the normal functions of these molecular targets supplies scorpions with a powerful mechanism of prey capture and, at the same time, a potent defensive weapon against competing conspecifics and predators [1,2,3,4]. Actually, which function came first in their evolutionary history is open to debate.

The classification schemes for scorpion toxins are various and depend on the criteria that are used. They can be classified according to their molecular size (or, rather, their number of constituent amino acid residues) into long and short chain toxins or according to their mechanism of action into neurotoxic and cytotoxic toxins [1,2,3,4]. Another method used to classify scorpion toxins is based on the presence or absence of discrete structural features—disulphide bridges—and the two groups are thus those that have these and those that do not [7]. The ion channel-interacting toxins universally fall into the first group.

In contrast to these, scorpion venom peptides without disulphide bridges constitute a minor fraction of the venom peptidome, and although only a few of these components have been isolated and biologically-characterised, nevertheless, they are diverse in both their structures and putative functions. Most of these peptide toxins are low molecular mass peptides of 13–50 amino acid residues and are either predominantly bradykinin-potentiating peptides (BPPs) or antimicrobial peptides (AMPs), while others may have protease inhibitory activity [7,8].

The AMPs from scorpion venom are usually cationic, amphipathic, α-helical peptides of low molecular mass (2–5 kD). They exhibit a broad-spectrum of activity against Gram-positive bacteria, Gram-negative bacteria and fungi, by causing membrane lysis. Some peptides, such as hadrurin, are highly potent against both Gram-positive bacteria and Gram-negative bacteria without an apparent preference, whereas others exhibit more selective activity against either Gram-negative bacteria (parabutoporin) or Gram-positive bacteria (IsCT and BmKn2) [9,10].

Here, we report two novel peptides isolated from the venom of the North African scorpion, *Androctonus aeneas*, that were shown to have more selective growth-inhibitory activities against the Gram-positive bacterium, *Staphylococcus aureus (S. aureas)*, and the yeast, *Candida albicans (C. albicans)*, than against the Gram-negative bacterium, *Escherichia coli (E. coli)*. The primary structures of these two novel AMPs, which were named AaeAP1 and AaeAP2, were established by MS/MS fragmentation sequencing using an electrospray ionisation (ESI) mass spectrometer. By isolating venom-derived polyadenylated mRNA, a cDNA library was constructed, and through the application of RACE PCR, the cDNAs encoding the biosynthetic precursors of both novel peptides were cloned. Both mature peptides, as predicted from the respective cDNA open-reading frames, were located in the same reverse-phase HPLC fraction of venom. Synthetic replicates of both natural AMPs were produced by solid-phase peptide synthesis, as were cationicity-, amphipathicity-enhanced analogues of each, named AaeAP1a and AaeAP2a, respectively. All four synthetic peptides were evaluated for antimicrobial, haemolytic and anticancer cell activities. The synthetic analogues, AaeAP1a and AaeAP2a, were, as expected, more potent in terms of their antimicrobial activities when compared to their corresponding templates, with only a small increase observed in haemolytic activity. In contrast, antiproliferative activity against a panel of human cancer lines was only observed for the analogue peptides and was absent for the natural templates.

## 2. Results

### 2.1. Identification and Structural Analysis of Mature Novel AMPs from Venom

**Figure 1:**
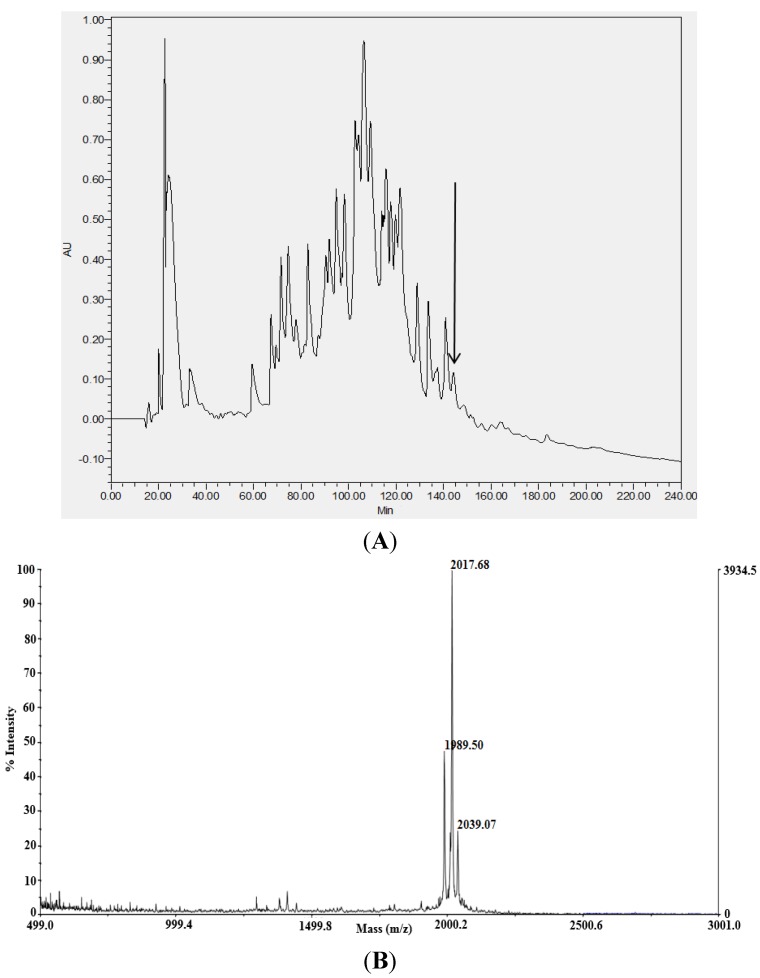
(**A**) Reverse-phase HPLC chromatogram of lyophilised *Androctonus aeneas* venom. The arrow indicates the elution position/retention time of the peptide fraction (#145) exhibiting antimicrobial activity. AU = 214 nm, and the gradient was formed from TFA/water (0.05:99.95, *v*/*v*) to TFA/water/acetonitrile (0.1:19.95:80.00, *v*/*v*/*v*) in 240 min at a flow rate of 1 mL/min. (**B**) MALDI-TOF mass spectrum of a sample of Fraction #145 indicating singly-charged [M + H]^+^ ions at *m*/*z* 1989.50 and *m*/*z* 2017.68. The ion at *m*/*z* 2039.07 is likely to be a sodium adduct ion [M + Na]^+^ of the peptide at *m*/*z* 2017.68.

Screening of the reverse-phase HPLC fractions of lyophilised *Androctonus aeneas* venom for antimicrobial activity resulted in the identification of significant antimicrobial activity in a single fraction, number 145 (Figure 1). Analysis of the doubly-charged ions of the two major peptides present in this fraction by MS/MS, with subsequent sequence calling based on fragment ion profiles, revealed the presence of two novel, structurally-related putative antimicrobial peptides. As the more readily-fragmented doubly-charged ions from each peptide were used for MS/MS fragmentation, the resultant spectra contained both singly- and doubly-charged fragment ions (Table 1 and Table 2).

### 2.2. Cloning of AMP Precursor-Encoding cDNAs

Two novel AMP-encoding cDNAs were consistently amplified and cloned from the cDNA library derived from the venom of the scorpion, *Androctonus aeneas*, and each encoded a single copy of their respective mature peptides (Figure 2). Translation of the open reading frames of these homologous AMP biosynthetic precursors demonstrated that each consisted of 74 amino acid residues and that the conserved prepro-region of each peptide precursor open-reading frame included a putative 22-amino acid residue signal peptide followed directly by the mature peptide sequences. The mature peptide sequences were flanked at their *C*-terminals by the post-translational α-amidation signal motif, -Gly-Arg-, indicating the high probability that each mature peptide sequence was *C*-terminally amidated. The peptides were named AaeAP1 and AaeAP2, in accordance with the accepted terminology (Aae = *Androctonus aeneas*; AP = antimicrobial peptide). The alignment of both peptides with homologues in the NCBI database is shown in Table 3. The nucleotide sequences of the precursor-encoding cDNAs of both peptides have been deposited in the European Molecular Biology Laboratory Nucleotide Sequence Database under the accession codes, HG792997 (AaeAP1) and HG792998 (AaeAP2).

**Table 1:** Predicted singly- and doubly-charged *b*-ions and *y*-ions arising from MS/MS fragmentation of AaeAP1. Ions observed following MS/MS fragmentation are indicated in bold typeface and are underlined.

#1	b(1+)	b(2+)	Sequence	y(1+)	y(2+)	#2
1	148.07570	74.54149	F	-	-	19
2	261.15977	131.08352	L	1869.13213	**935.06970**	18
3	**408.22819**	204.61773	F	1756.04806	**878.52767**	17
4	**495.26022**	248.13375	S	1608.97964	**804.99346**	16
5	**608.34429**	304.67578	L	1521.94761	761.47744	15
6	**721.42836**	361.21782	I	1408.86354	**704.93541**	14
7	**818.48113**	**409.74420**	P	1295.77947	**648.39337**	13
8	**905.51316**	453.26022	S	1198.72670	599.86699	12
9	1004.58158	**502.79443**	V	1111.69467	**556.35097**	11
10	**1117.66565**	**559.33646**	I	1012.62625	506.81676	10
11	**1188.70277**	594.85502	A	899.54218	450.27473	9
12	**1245.72424**	623.36576	G	828.50506	414.75617	8
13	**1358.80831**	**679.90779**	L	771.48359	386.24543	7
14	**1457.87673**	729.44200	V	658.39952	**329.70340**	6
15	**1544.90876**	772.95802	S	559.33110	280.16919	5
16	**1615.94588**	808.47658	A	472.29907	236.65317	4
17	**1729.02995**	865.01861	I	401.26195	201.13461	3
18	1885.13107	**943.06917**	R	288.17788	144.59258	2
19	-	-	*C*-Amidated	132.07676	66.54202	1

**Table 2:** Predicted singly- and doubly-charged *b*-ions and *y*-ions arising from MS/MS fragmentation of AaeAP2. Ions observed following MS/MS fragmentation are indicated in bold typeface and are underlined.

#1	b(1+)	b(2+)	Sequence	y(1+)	y(2+)	#2
1	148.07570	74.54149	F	-	-	19
2	261.15977	131.08352	L	1841.10083	**921.05405**	18
3	**408.22819**	204.61773	F	1728.01676	**864.51202**	17
4	**495.26022**	248.13375	S	**1580.94834**	**790.97781**	16
5	**608.34429**	304.67578	L	**1493.91631**	747.46179	15
6	**721.42836**	361.21782	I	**1380.83224**	690.91976	14
7	**818.48113**	**409.74420**	P	**1267.74817**	**634.37772**	13
8	**905.51316**	**453.26022**	S	**1170.69540**	585.85134	12
9	**976.55028**	488.77878	A	**1083.66337**	**542.33532**	11
10	**1089.63435**	**545.32081**	I	**1012.62625**	506.81676	10
11	**1160.67147**	**580.83937**	A	**899.54218**	450.27473	9
12	**1217.69294**	**609.35011**	G	**828.50506**	414.75617	8
13	**1330.77701**	665.89214	L	**771.48359**	386.24543	7
14	**1429.84543**	715.42635	V	**658.39952**	**329.70340**	6
15	**1516.87746**	758.94237	S	**559.33110**	280.16919	5
16	**1587.91458**	794.46093	A	**472.29907**	236.65317	4
17	**1700.99865**	851.00296	I	**401.26195**	201.13461	3
18	1857.09977	**929.05352**	R	**288.17788**	144.59258	2
19	-	-	*C*-Amidated	132.07676	66.54202	1

**Figure 2:**
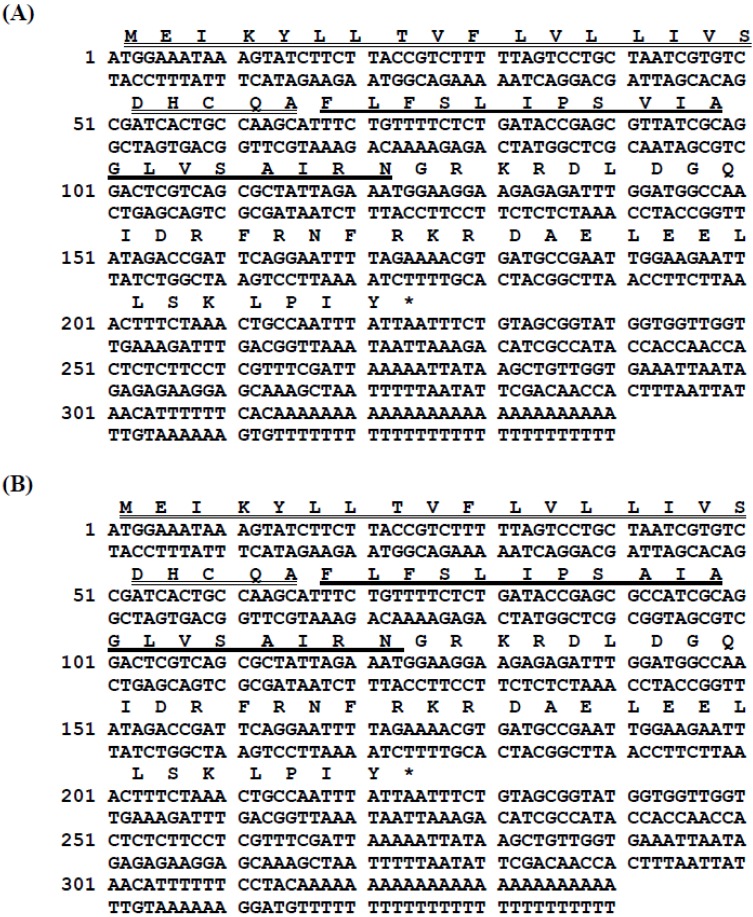
Nucleotide and translated open-reading frame amino acid sequences of cloned cDNAs encoding the biosynthetic precursors of AaeAP1 (**A**) and AaeAP2 (**B**). Putative signal peptides are double-underlined; mature peptides are single-underlined; and stop codons are indicated by asterisks.

### 2.3. Secondary Structure Prediction and Physiochemical Properties of AaeAP1 and -2 and Their Cationicity-/Amphipathicity-Enhanced Analogues, AaeAP1a and -2a

**Figure 3:**
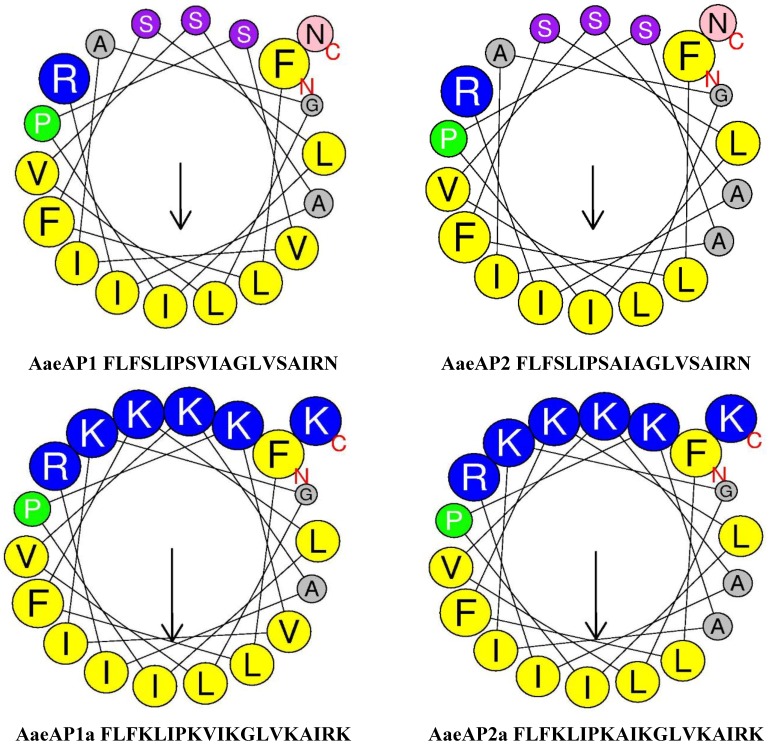
Helical wheel plots of AaeAP1, AaeAP2, AaeAP1a and AaeAP2a. Note the increased amphipathicity and cationicity that have been engineered into the analogues, AaeAP1a and AaeAP2a.

The data obtained through the application of robust web-based programmes for secondary structure prediction and assessment of the additional physicochemical properties of both synthetic replicates of the natural peptides and their engineered cationicity-enhanced analogues are summarised in Table 4, and helical wheel plots are illustrated in Figure 3, respectively.

**Table 3:** Alignment of the primary structures of the novel antimicrobial peptides, AaeAP1 and AaeAP2, with scorpion venom homologues present in the NCBI database. Note that AaeAP1 and AaeAP2 are both nonadecapeptides, and the majority of homologues are octadecapeptides. Note also that all peptides are *C*-terminally amidated (a). Identical residues are represented by dashes.

Species	Peptide	Primary structure	Nucleotide sequence database
*Androctonus aeneas*	AaeAP1	FLFSLIPSVIAGLVSAIRNa	(Accession No. HG792997)
*Androctonus aeneas*	AaeAP2	--------A----------a	(Accession No. HG792998)
*Mesobuthus martensii*	Kb1	--------A-S--I--FKa	(Accession No. AF159979)
*Mesobuthus eupeus*	caerin-like AMP	--------A-S--I--FKa	(Accession No. KC108907)
*Androctonus amoreuxi*	AamAP1	-------HA-G--I--FKa	(Accession No. FR821613)
*Isometrus maculatus*	imcroporin	-F---L--L-G------Ka	(Accession No. FJ750949)
*Lychas mucronatus*	mucroporin	--G----L-G-----FKa	(Accession No. EU669864)
*Mesobuthus martensii*	BmKb2	--S-----A-S--I--FKa	(Accession No. AF543048)
*Tityus costatus*	AMP clone 5	-F------L-G---F--Ka	(Accession No. AY740687)
*Androctonus amoreuxi*	AamAP2	-P-----HA-GG-ISAIKa	(Accession No. FR821614)

**Table 4:** Secondary structure predictions and some physiochemical properties of AaeAPs and their cationicity-enhanced analogues described in this study. Net charges were calculated at pH 7. The random coil is represented by c, the extended strand by e and the “helix” by h.

Peptides	Secondary structure	α-Helix (%)	Hydrophobicity (H)	Hydrophobic moment (μH)	Net charge
AaeAP1	FLFSLIPSVIAGLVSAIRN cccccccchhhheeeeeec	21.05	0.849	0.455	+2
AaeAP2	FLFSLIPSAIAGLVSAIRN cccccccchhhheeeeeec	21.05	0.801	0.428	+2
AaeAP1a	FLFKLIPKAIKGLVKAIRK ccceecchhhhhheeeeec	31.58	0.610	0.669	+7
AaeAP2a	FLFKLIPKVIKGLVKAIRK ccceecchhhhhheeeeec	31.58	0.562	0.641	+7

### 2.4. Antimicrobial/Haemolytic Activities of AaeAP1 and -2 and Their Cationicity-/Amphipathicity-Enhanced Analogues, AaeAP1a and -2a

The data obtained from both MIC and MBC assays of the four synthetic peptides (natural and analogues) are summarised in Table 5 and Table 6. AaeAP1 and AaeAP2 displayed identical MICs against *S. aureus* (16 mg/L) and *C. albicans* (32 mg/L), respectively, but both were ineffective against *E. coli* up to and including the highest concentrations tested (512 mg/L). Their MBCs were found to be generally double that of their MICs at 32 mg/L for *S. aureus* with AaeAP1 and 64 mg/L for *C. albicans* with both synthetic natural peptides. However, the MBC for AaeAP2 against *S. aureus* remained the same as its MIC. AaeAP2 was found to be four-fold less haemolytic than AaeAP1, requiring 64 mg/L rather than 16 mg/L to effectively completely lyse the red blood cells employed in the model assay. The biological behaviour of the two synthetic analogues was found to be quite different to that of the synthetic natural peptides. The MICs of both analogues were both found to be four-fold lower against *S. aureus* (4 mg/L) and eight-fold lower against *C. albicans* (4 mg/L). The most dramatic observation, however, was that both analogues exhibited a potent action against *E. coli* with identical MICs of 16 mg/L. The haemolytic activity of AaeAP1 was reduced two-fold by cationicity/amphipathicity enhancement to 32 mg/L, but that of AaeAP2 remained unchanged at 64 mg/L.

**Table 5:** Minimum inhibitory concentrations (MICs) and minimum bactericidal concentrations (MBCs) of synthetic AaeAP1 and AaeAP2 and their cationicity/amphipathicity analogues, AaeAP1a and AaeAP2a; NT, not tested.

Peptides	MIC (mg/L)			MBC (mg/L) 100% hemolysis (mg/L)			
	*S. aureus*	*E. coli*	*C. albicans*	*S. aureus*	*E. coli*	*C. albicans*	Horse red cells
AaeAP1	16	>512	32	32	NT	64	16
AaeAP2	16	>512	32	16	NT	64	64
AaeAP1a	4	16	4	32	32	16	32
AaeAP2a	4	16	4	32	32	16	64

**Table 6:** Structures of natural peptides and their cationicity-/amphipathicity-enhanced analogues.

Peptide	Primary structure	Mass (Da)
AaeAP1	FLFSLIPSVIAGLVSAIRNamide	2016.18
AaeAP2	FLFSLIPSAIAGLVSAIRNamide	1986.15
AaeAP1a	FLFKLIPKVIKGLVKAIRKamide	2209.48
AaeAP2a	FLFKLIPKAIKGLVKAIRKamide	2181.45
	* * * * *	

* Sites of cationicity enhancement using lysyl (K) residue substitution for Ser(S)^4^, Ser(S)^8^, Ala(A)^11^ Ser(S)^15^ and Asn(N)^19^. Net positive charge increase from +2 (natural peptides) to +7 (respective analogues).

### 2.5. The Antiproliferative Effects of AaeAP1 and -2 and Their Cationicity-/Amphipathicity-Enhanced Analogues, AaeAP1a and -2a, against a Panel of Human Cancer Cell Lines

**Figure 4:**
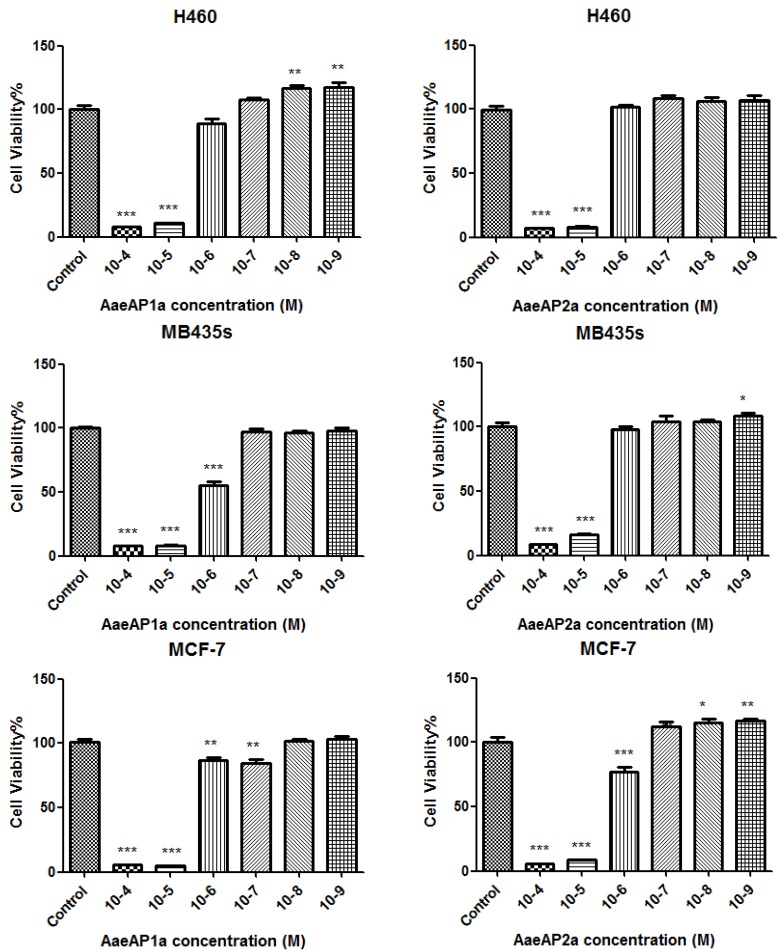

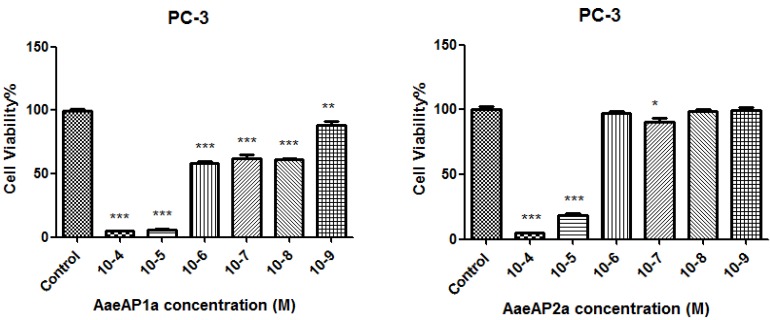
Dose-dependent antiproliferative effects of the analogues, AaeAP1a and AaeAP2a, on the human cancer cell lines, H460, MB435s, MCF-7 and PC3, after 24 h of incubation. The levels of significance are: * *p* < 0.05; ** *p* < 0.01; *** *p* < 0.001. Note that at low concentrations (10^−9^ M and 10^−8^ M), AaeAP1a significantly increased the proliferation of H460 cancer cells (*p* < 0.01). AaeAP2a significantly increased (*p* < 0.05) the proliferation of MB435s cancer cells at 10^−9^ M and the proliferation of MCF-7 cancer cells at 10^−8^ M (*p* < 0.05) and at 10^−9^ M (*p* < 0.01). The growth inhibition of cells at each peptide concentration was calculated as a percentage of the growth observed in controls, which was treated as 100%.

The ability of the four peptides to inhibit the proliferation of four different human cancer cell lines, at concentrations ranging between 10^−4^ and 10^−9^ M, was assessed using the MTT assay. The synthetic replicates of the natural peptides exhibited no activity on the proliferation of all of the human cancer cell lines tested at the concentrations employed. In contrast, both synthetic cationic-/amphipathicity-enhanced analogues displayed antiproliferative activity against all four cell lines (Figure 4).

## 3. Discussion

The earliest scorpion fossils date back to the Silurian Period some 430 million years ago, and these possess the typical telson that, in modern scorpions, is the organ that synthesises, stores and delivers venom [11]. Scorpion venoms have thus been subjected to aeons of natural selection, resulting in optimisation of the component toxins for molecular targets within their predominantly insect prey to facilitate rapid and effective immobilisation prior to ingestion [2,3,12,13]. This prerequisite rapid mode of action has resulted in the evolution of an array of neuromuscular toxins that target many types of ion channels in the membranes of excitable cells [2,3].

Although primarily directed against molecular targets in insect prey, some of the component neurotoxins are also effective on predators and can be used defensively. Ion channel proteins are functionally fundamental to the normal physiology of all cells, and often, sites of toxin action on specific molecular targets are conserved across diverse and distant taxa, such that a small number of scorpions possess venoms that are toxic to humans [14,15]. However, of the approximately 1,700 species of scorpions, only around 30 species have venoms that are considered to be so [2]. Nevertheless, this small number of species is of widespread global distribution and causes significant human morbidity and mortality, which is one of the major reasons why scorpion venom research is so important [2,3,4,16]. In view of the fact that scorpion envenomation can be lethal, even within developed countries, such as the U.S., the major focus of research on scorpion venom has been on the lethal components: the ion channel toxins [15,16,17,18]. Two additional peptides, DLDGQIDRFRNFR and DAELEELLSKLPIY, falling between the -KR-processing sites and/or the *C*-terminus within both precursors, were not detected in the reverse-phase HPLC fractions of venom, but were synthesized and subjected to the bioassays described here, in which they were found to be inactive (data not shown).

However, scorpion venom contains a significant number of peptides that do not act on ion channels and whose precise biological actions remain obscure. One of these groups are the so-called antimicrobial peptides [19], and little attention was paid to these until the isolation of the prototypes, hadrurin and scorpine, from the venoms of the scorpions, *Hadrurus aztecus* and *Pandinus imperator*, respectively [20,21]. Since then, several other AMPs with a broad spectrum of action have been isolated from the venoms of other scorpions [22,23]. Since some scorpions had been observed to spray venom on their own bodies to clean invading microorganisms, these venom AMPs were assumed as defence tools against infection [7,20,21]. This hypothesis was further supported by the observation of the upregulation of transcription of AMPs within the bacterially-challenged venom glands of the scorpion, *Buthus martensii* [24]. However, in common with many other arthropod taxa, scorpions possess classical potent broad-spectrum defensins in their haemolymph, and the production of these from haemocytes is induced upon infection [25]. These defensins are ancient molecules, as evidenced by the finding of highly-conserved structural homologues in ticks, mussels and even fungi [26].

The present study describes the molecular cloning of two putative AMP-encoding cDNAs from a venom-derived cDNA library of the scorpion, *Androctonus aeneas*, with subsequent identification of the mature AMPs in venom fractions, their chemical synthesis and determination of their antimicrobial spectra and potencies. The AMPs were both *C*-terminally-amidated 19-mers and exhibited moderately potent activity against a standard Gram-positive bacterium and a yeast, but were ineffective against a standard Gram-negative bacterium. *C*-terminal amidation is a common feature of bioactive peptides found in many taxa across the evolutionary tree and is thought to act in eliminating the usual *C*-terminal negative charge, thus creating a more hydrophobic character in this region that facilitates peptide interaction, either specifically or non-specifically with their membrane target [27]. As endogenous scorpion defensins are much more potent and to have a broad spectrum in their actions, these venom-derived AMPs may not function primarily as anti-infective agents, but may have another primary biological role. Nevertheless, due to their low molecular masses and ease of chemical synthesis, they provide the medicinal chemist with natural templates for the design of analogues with the view of potentially improving both their potencies and spectra of action through rational structural engineering.

As a result of many studies on the mode of action of AMPs, it has become clear that a few fundamental physicochemical features, namely the degree of cationicity and amphipathicity, are known to be important for functionality. For this reason, we engineered analogues of each AMP with enhanced cationic and amphipathic features. To this end, helical wheel plots were constructed for each natural AMP template to determine the distribution of individual amino acid residue chains; then, several were substituted by Lys (K) residues, and the plots were reconstructed. The two analogues so designed had clear and unambiguous amphipathicity, as shown in these latter plots, and there was an associated significant increase in their overall net positive charges. Of course, such plots are predictive, and the resulting engineered peptides were then subjected to a bioassay to determine if such modifications had produced any changes in the biological activities.

As expected, the MICs of both analogue peptides against *S. aureus* and *C. albicans* decreased four-fold (16 > 4 mg/L) and eight-fold (32 > 4 mg/L), respectively. However, both analogues were found to possess a potent activity against *E. coli* (16 mg/L), an activity that was not noted with the synthetic natural peptide templates at concentrations up to and including 512 mg/L. The MBC observed for both synthetic natural peptides against *S. aureus* remained essentially unchanged for the analogues, but decreased four-fold against *C. albicans* (64 > 16 mg/L). The MBC for *E. coli* obtained with both analogues was found to be 32 mg/L. Despite these observed increases in antimicrobial potencies and the broadening of the spectrum of actions, the analogue peptides either remained unchanged in haemolytic potential (AaeAP2a, 64 mg/L) or exhibited a reduction in such a potential (AaeAP1a, 16 > 32 mg/L). These cytotoxic indices remained above their respective MIC values, particularly for *S aureus* and *C. albicans*. Thus, the engineering of these cationicity- and amphipathicity-enhanced analogues from both natural peptide templates resulted in clear increases in both their antimicrobial potencies and spectrum of activity, while leaving their potential for mammalian cell cytotoxicity relatively unchanged or slightly reduced.

Several previous studies on antimicrobial peptides from a variety of natural sources have reported that some possess antiproliferative activity on human cancer cell lines [28]. This effect undoubtedly increases the putative therapeutic potential of such peptides and could provide a novel means of treating human cancers [28]. The synthetic natural peptides, AaeAP1 and AaeAP2, described here, were found to be inactive for inhibiting the proliferation of four different human cancer cell lines. However, their respective cationicity- and amphipathicity-enhanced analogues were both found to possess a dose-dependent inhibition of all four cell lines. The engineered increase in net positive charge in both analogues probably facilitated a greater interaction with target cell membranes. This effect is clearly observed by the increased potency in the antimicrobial effects and appears with both analogues for cancer cells. It could be that the optimum micellar concentration for cancer cell membrane perturbation is exceeded for the analogues, but sub-optimal for the natural peptides. This is an observation that deserves further study. At a concentration of 10^−5^ M (equivalent to approximately 22 mg/L), both peptides caused more than 85% inhibition of the proliferation of cells. However, their effects were somewhat more selective on certain cell lines at much lower concentrations, indicating that a general cytotoxic effect was not responsible for all of the observed effects. For instance, a significant inhibition of the proliferation of the human prostate cancer cell line, PC3 (*p* < 0.001 compared to the controls), was observed at a concentration of 10^−9^ M (1 nM–2.2 µg/L), at which concentration there was no observable haemolytic effects. Of additional interest were the observations with the other three cell lines at these sub-micromolar concentrations, where a significant enhancement of proliferation, ranging from *p*-values of <0.05 through 0.001, was observed for both analogues. Antimicrobial peptides are thought to act predominantly through membrane lysis of both microbial and cancer cells, due to their similarities in membrane compositions, which are different from those of normal eukaryotic cells [26,28]. However, there is recent evidence to suggest that other, more subtle mechanisms may be involved, which include effects on multiple intracellular targets [28,29,30]. This could be at concentrations lower than those that cause cytolysis, and increasing membrane permeability at sub-lytic concentrations affords an increase in proliferative potential, due to some means as simple as increasing access to nutrients or growth factors in the culture medium or by some other more complex route, such as a positive modulation of discrete signalling pathways.

## 4. Experimental Section

### 4.1. Acquisition of Scorpion Venom

A 10-mg sample of authentic lyophilised *Androctonus aeneas* venom was obtained from Latoxan, Valence, France. Donor scorpions were collected in the field and identified by experts before relocation to the company facility in France. Venom was collected by trained individuals using electrical stimulation (15 V) consistent with obtaining sufficient material, but also with causing a low degree of damage to the animals. Venom samples were obtained by this procedure at regular intervals for considerable periods of time (Latoxan, personal communication). All procedures using live animals had been approved by appropriate national licensing and ethics authorities, and permits to acquire animals from the wild had been secured from appropriate national bodies prior to the collection and relocation of specimens.

### 4.2. Isolation and Structural Characterisation of Antimicrobial Activity from Lyophilised Venom

A 5-mg sample of lyophilised *Androctonus aeneas* venom was dissolved in 0.5 mL of trifluoroacetic acid (TFA)/water (0.05/99.95; *v*/*v*) and clarified by centrifugation (1100× *g*; 5 min). The supernatant was decanted and subjected to reverse-phase HPLC fractionation using a Waters HPLC system (Worcester County, MA, USA) fitted with an analytical column (Phenomenex C-5; 250 mm × 4.6 mm). This was eluted with a linear gradient formed from TFA/water (0.05/99.95; *v*/*v*) to TFA/water/acetonitrile (0.05/19.95/80.00; *v*/*v*/*v*) in 240 min at a flow rate of 1 mL/min. The column effluent was continuously monitored at λ 214 nm, and fractions (1 mL) were automatically collected at minute intervals. Samples (100 μL) were removed from each fraction, lyophilised and stored at −20 °C prior to being subjected to the antimicrobial assay. The molecular masses of the peptides in each fraction displaying antimicrobial activity were analysed using matrix-assisted, laser desorption/ionisation, time-of-flight mass spectrometry (MALDI-TOF MS) on a linear time-of-flight Voyager DE mass spectrometer (Perseptive Biosystems, Framingham, MA, USA) in positive detection mode, using α-cyano-4-hydroxycinnamic acid as the matrix. The amino acid sequences of the resolved peptides in the active antimicrobial fraction were determined by MS/MS fragmentation sequencing using an LCQ-Fleet electrospray, ion-trap mass spectrometer (Thermo Fisher Scientific, San Jose, CA, USA).

### 4.3. Antimicrobial Screening Assays

The standard panel of microorganisms used for activity screening were the Gram-positive bacterium, *Staphylococcus aureus* (*S. aureus*) (NCTC 10788), the Gram-negative bacterium, *Escherichia coli* (*E. coli*) (NCTC 10418), and the yeast, *Candida albicans* (*C. aibican*) (NCPF 1467). All are used routinely for such purposes and are non-pathogenic strains. The antimicrobial activity of fraction samples was initially evaluated using a simple inhibition zone assay on Luria–Bertani (LB)-agarose plates [31]. To study the microbicidal effects, each fraction sample, following lyophilisation and reconstitution in phosphate-buffered saline (PBS), was added to a 2 mm-diameter hole punched in the surface of the agar plate. The plates were then incubated at 37 °C overnight.

### 4.4. Molecular Cloning of Antimicrobial Peptide Biosynthetic Precursor-Encoding cDNAs from the Venom-Derived cDNA Library

A 5-mg sample of lyophilised venom was dissolved in 1 mL of cell lysis/mRNA protection buffer supplied by Dynal Biotech, Merseyside, UK, and polyadenylated mRNA was isolated by magnetic oligo-dT beads, as described by the manufacturer (Dynal Biotech). The isolated mRNA was subjected to 5'- and 3'-rapid amplification of cDNA ends (RACE) procedures to obtain full-length antimicrobial peptide precursor nucleic acid sequence data using a SMART-RACE kit (Clontech, Oxford, UK), essentially as described by the manufacturer. Briefly, the 3'-RACE reactions employed a nested universal primer (NUP) (supplied with the kit) and a degenerate sense primer pool (S1: 5'-TTYHTITTYWSNHTIHTICC-3') (Y = C/T, H = A/T/C, I = deoxyinosine, W = A/T, S = C/G, N = A/C/T/G) that was complementary to the amino acid sequence, F-L/I-F-S-L/I-L/I-P-, of the *N*-terminal region of the scorpion venom AMPs. The 3'-RACE reactions were purified and cloned using a pGEM-T vector system (Promega Corporation, Southampton, UK) and sequenced using an ABI 3100 automated sequencer. The sequence data obtained from these 3'-RACE products were used to design a specific antisense primer (AS: 5'-CGAAACGAGGAAGAGAGACCAA-3') to a conserved site within the 3'-non-translated region of these cDNAs. 5'-RACE was carried out using this specific primer in conjunction with the NUP RACE primer, and the resultant products were purified, cloned and sequenced.

### 4.5. Prediction of Putative AMP Secondary Structures and Physicochemical Properties

Putative peptide secondary structures were predicted using the GOR secondary structure prediction method software (Version 2.2.26, Pole Bioinformatique Lyonnais, Lyon, France, 2014) (http://npsa-pbil.ibcp.fr/cgi-bin/npsa_automat.pl?page=npsa_gor4.html). Additional physicochemical properties, including hydrophobicity, hydrophobic moments, net charge at neutral pH and helical wheel plots, were determined by using the Heliquest server on-line (http://heliquest.ipmc.cnrs.fr/cgi-bin/ComputParamsV2.py) [32].

### 4.6. Peptide Synthesis and Purification

The two natural antimicrobial peptides and their cationicity-/amphipathicity-enhanced analogues were each separately synthesised by a solid-phase methodology using Rink amide resin and standard Fmoc chemistry, by means of an automated PS3 peptide synthesiser (Protein Technologies, Tucson, AZ, USA), followed by deprotection and cleavage from the resin. Each synthetic peptide was analysed by both reverse-phase HPLC and MALDI-TOF mass spectrometry to establish the degree of purity and authenticity.

### 4.7. Antimicrobial Minimal Inhibitory Concentration and Minimum Bactericidal Assays

The antimicrobial activity of each synthetic peptide was assessed by means of determining the minimal inhibitory concentrations (MICs) against model strains of Gram-positive bacteria, *S. aureus*, Gram-negative bacteria, *E. coli*, and yeast, *C. albicans*, respectively. The model microorganisms were initially incubated in Mueller–Hinton broth (MHB) for 16–20 h. Upon achieving their respective logarithmic growth phases, as measured by the optical density (OD) of media at 550 nm, the cultures were diluted to 1 × 10^6^ colony-forming units (cfu)/mL for the bacteria and to 5 × 10^5^ cfu/mL for the yeast. Samples of these were then added to 96-well microtitre plates and mixed with the tested peptides at various concentrations (1–512 mg/L). After 24 h of incubation, the OD of each well was measured at 550 nm using a Synergy HT plate reader (BioTek, Winooski, VT, USA), and the data were analysed using GraphPad Prism 5 software. The MIC was defined as the minimum concentration of peptide with an OD the same as the negative control (medium minus microorganisms). After this, 10 μL of the medium from each well were inoculated onto a Mueller–Hinton agar (MHA) plate and incubated for 24 h for the measurement of minimum bactericidal concentrations (MBCs), which were defined as the concentrations of peptide from which no colonies could be grown.

### 4.8. Haemolysis Assay

A 2% (*v*/*v*) suspension of red blood cells was prepared from defibrinated horse blood (TCS Biosciences Ltd., Buckingham, UK). Peptide solutions at different concentrations (1–512 mg/L) were prepared as described in the previous section. Red blood cell suspension samples (200 μL) were incubated with the range of peptide concentrations as defined before, at 37 °C for 60 min and 120 min. Lysis of red cells was assessed by measurement of optical density at λ = 550 nm using an ELISA plate reader (Biolise BioTek EL808, Winooski, VT, USA). The negative controls employed consisted of a 2% (*v*/*v*) red cell suspension and sodium phosphate-buffered saline (PBS) in equal volumes, and the positive controls consisted of a 2% (*v*/*v*) red cell suspension and an equal volume of PBS containing 2% (*v*/*v*) of the non-ionic detergent, Triton X-100 (Sigma-Aldrich, St. Louis, MO, USA).

### 4.9. Culture and Maintenance of Human Cancer Cell Lines

The human prostate carcinoma cell line, PC-3, and the human lung adenocarcinoma cell line, NCI-H460, were cultured using RPMI-1640 culture medium (Invitrogen, Paisley, UK). Before culturing the cells, this medium was supplemented with 10% (*v*/*v*) foetal bovine serum (FBS) (Sigma-Aldrich, St. Louis, MO, USA) and 0.1% (*w*/*v*) gentamicin (Sigma-Aldrich, St. Louis, MO, USA), and the cells were seeded into 150-cm^2^ culture flasks (Nunc, Roskilde, Denmark). The human breast carcinoma cell line, MDA-MB-435s, and the non-tumourigenic mammary gland cell line, MCF-7, were cultured using a Dulbecco’s modified eagle’s medium (DMEM) (Sigma-Aldrich, St. Louis, MO, USA), which was supplemented with 10% FBS and 0.1% gentamicin. The cells were seeded into 150-cm^2^ culture flasks.

### 4.10. Assessment of Anti-Proliferative Effects of AMPs on Human Cancer Cells Using the MTT Cell Viability Assay

Yellow-coloured MTT (3-(4,5-dimethylthiazol-2-yl)-2,5-diphenyltetrazolium bromide) is reduced by the mitochondria of living cells to form a purple-coloured (formazan) crystalline derivative. These crystals can be solubilised by the addition of dimethyl sulphoxide (DMSO) (Sigma-Aldrich, St. Louis, MO, USA) and their concentration determined spectrophotometrically, as detailed below.

The protocol employed has been described in detail previously [33]. Briefly, each cell line was seeded at a density of 5 × 10^3^ cells per well onto 96-well plates. After this, cells were treated with various concentrations of peptides (10^−9^–10^−4^ M) or with serum-free medium alone (*n* = 8 for each) and were incubated for 24 h. Following this, 10 μL of a 5-mg/mL MTT solution was added to each well and the plates incubated again for 4 h. The growth medium was later removed using a 1-mL syringe fitted with a 21-guage needle, and 100 μL of DMSO were added to each well and mixed vigorously to dissolve the formazan crystals that had developed. The absorbance was measured using an ELx808™ Absorbance Microplate Reader (BioTek, Winooski, VT, USA) at 550 nm, and the statistical analyses were performed using the Student’s *t*-test through GraphPad Prism software for Windows. The results were considered to be statistically significant if the *p*-value was <0.05. Growth inhibition of cells at each peptide concentration was calculated as the percentage of the growth observed in controls, which was treated as 100%.

## 5. Conclusions

Two novel 19-mer antimicrobial peptides were identified in the venom of the North African scorpion, *Androctonus aeneas*. Synthetic replicates of the novel natural peptides displayed antimicrobial activity against the Gram-positive bacterium, *S. aureus*, and the yeast, *C. albicans*, but were ineffective against the Gram-negative bacterium, *E. coli*, and at inhibiting the proliferation of four human cancer cell lines. In contrast, analogues of each, engineered for enhanced cationicity and amphipathicity, exhibited higher potencies against *S. aureus* and *C. albicans* and exhibited a high potency against *E. coli*. In addition, both analogues were effective at inhibiting the proliferation of all four human cancer cell lines with micromolar potencies and some with nanomolar potencies. These data illustrate the potential for the rational design of effective antimicrobial and anticancer peptides from natural peptide templates. Although preliminary in nature, the data presented the effective and selective actions of such designed peptide analogues on human cancer cells, and this is an area that warrants further and more systematic study.
